# Engineering strategies for apoptotic bodies

**DOI:** 10.1002/SMMD.20240005

**Published:** 2024-07-14

**Authors:** Zheyuan Hu, Shutong Qian, Qiuyu Zhao, Bolun Lu, Qian Lu, Yuhuan Wang, Liucheng Zhang, Xiyuan Mao, Danru Wang, Wenguo Cui, Xiaoming Sun

**Affiliations:** ^1^ Department of Plastic and Reconstructive Surgery Shanghai Ninth People's Hospital Shanghai Jiao Tong University School of Medicine Shanghai China; ^2^ Department of Plastic Surgery The First Affiliated Hospital College of Medicine Zhejiang University Hangzhou China; ^3^ Department of Orthopaedics Shanghai Key Laboratory for Prevention and Treatment of Bone and Joint Diseases Shanghai Institute of Traumatology and Orthopaedics Ruijin Hospital Shanghai Jiao Tong University School of Medicine Shanghai China

**Keywords:** apoptotic bodies, engineering, extracellular vesicles

## Abstract

Extracellular vesicles (EVs) are lipid bilayer vesicles containing proteins, lipids, nucleic acids, and metabolites secreted by cells under various physiological and pathological conditions that mediate intercellular communication. The main types of EVs include exosomes, microvesicles, and apoptotic bodies (ABs). ABs are vesicles released during the terminal stages of cellular apoptosis, enriched with diverse biological entities and characterized by distinct morphological features. As a result, ABs possess great potential in fields like disease diagnosis, immunotherapy, regenerative therapy, and drug delivery due to their specificity, targeting capacity, and biocompatibility. However, their therapeutic efficacy is notably heterogeneous, and an overdose can lead to side effects such as accumulation in the liver, spleen, lungs, and gastrointestinal system. Through bioengineering, the properties of ABs can be optimized to enhance drug‐loading efficiency, targeting precision, and multifunctionality for clinical implementations. This review focuses on strategies such as transfection, sonication, electroporation, surface engineering, and integration with biomaterials to enable ABs to load cargoes and enhance targeting, providing insights into the engineering of ABs.

## Graphical abstract

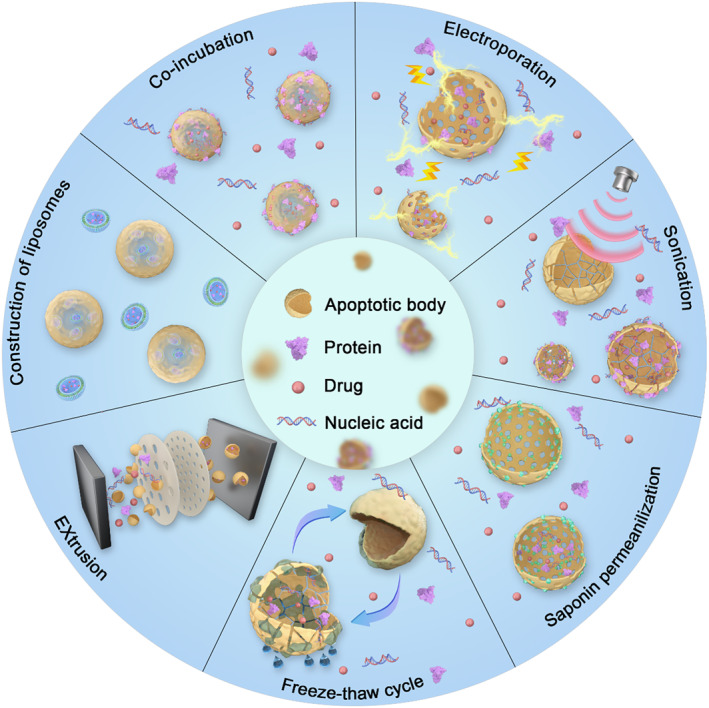

**Key points**

- The entire process of apoptotic bodies (ABs) from induction, isolation, application, and modification is introduced.
- The advantages and disadvantages of each of the engineering strategies for ABs are discussed.
- The prospects and challenges of ABs are discussed.

## 1 INTRODUCTION

Cells secrete extracellular vesicles (EVs) with proteins, lipids, nucleic acids, and metabolites that mediate intercellular communication under various conditions.^1^, ^2^, ^3^ EVs are conventionally categorized based on their diameter and intracellular origin, falling into three primary classifications: exosomes, microvesicles, and apoptotic bodies (ABs).^4^, ^5^ Exosomes are the smallest type of EVs, with a size ranging from 30 to 100 nm, originating from multivesicular bodies (MVBs). Microvesicles range in size from 50 to 1000 nm and directly stem from the cell plasma membrane. The applications of exosomes and microvesicles are primarily concentrated in disease diagnosis, cancer treatment, vaccination, immunotherapy, regenerative therapy, and drug delivery.^6^, ^7^, ^8^, ^9^, ^10^, ^11^ Exosomes, in particular, have been widely used due to their high biocompatibility and bioengineering operability.^12^, ^13^, ^14^, ^15^ Nonetheless, challenges such as non‐specific targeting, intricate preparation procedures, and inconsistent therapeutic efficacy underscore the imperative for novel methodologies in extracellular vesicle therapeutics.

**FIGURE 1:**
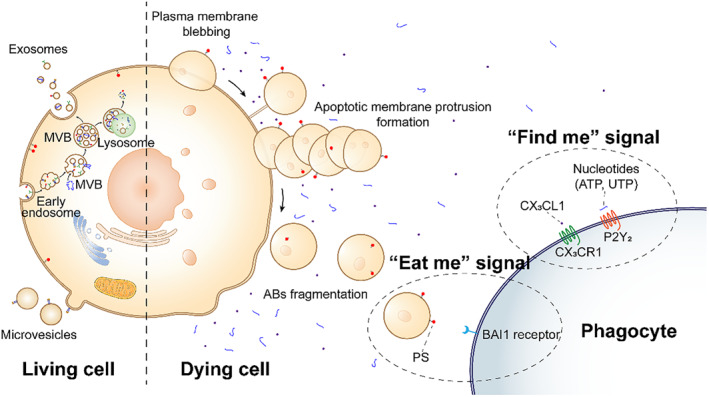
Schematic representation of the biogenesis and secretion of EVs and the “find‐me” and “eat‐me” signals. Exosomes originate from the inward budding of endosomal membranes and accumulate in MVBs. Some MVBs undergo direct lysosomal degradation, while others fuse with the plasma membrane to release exosomes. Microvesicles are formed directly by the budding of the cell membrane. ABs are produced when cells undergo a highly regulated series of processes such as plasma membrane blebbing, apoptotic membrane protrusion formation, and AB fragmentation during the final stages of apoptosis. “Find‐me” signals: Apoptotic cells release mediators such as ATP, UTP, and CX3CL1, establishing a chemotactic gradient to attract phagocytes. “Eat‐me” signals: PS on the apoptotic body surface binds to phagocytic cell receptors, inducing phagocytosis.

Apoptotic extracellular vesicles (ApoEVs) are generated during the terminal phases of cell apoptosis following a stringently regulated series of morphological transformations. Larger ApoEVs, commonly referred to as ABs, typically exhibit diameters ranging from 1000 to 5000 nm, as revealed by optical and electron microscopic studies.^16^, ^17^, ^18^, ^19^, ^20^ The contents of ABs encompass nucleic acids, proteins, lipids, and other biomolecules, and these biomolecules can be transferred to other cells via phagocytosis or through the activation of the “eat me” signaling pathway, facilitated by the externalized phosphatidylserine (PS) on the cellular membrane.^21^, ^22^, ^23^, ^24^ When compared to exosomes and microvesicles, ABs offer several advantages: ease of preparation and purification, enhanced targeting specificity, and their larger size to carry more biomolecules and drugs. However, the inherent variability in the number, composition, and size of ABs renders their study considerably challenging. Thus, the pace of research into the mechanisms and applications of ABs has not been kept abreast with that of exosomes and microvesicles presently.^25^, ^26^ (Figure 1).

ABs possess significant therapeutic potential and are currently utilized in various domains such as inflammation modulation, bioactive molecule and drug delivery, regenerative medicine, and disease diagnostics. However, the therapeutic outcomes of systemic or localized injections of unmodified ABs are often suboptimal, with challenges such as insufficient targeting specificity, rapid clearance from circulation, and low bioavailability in vivo.^27^ Consequently, the engineering of ABs has garnered widespread attention, as such modifications can rectify these inherent limitations while amplifying their therapeutic advantages. This article provides a comprehensive review of the induction, purification, and engineering techniques of ABs including transfection, electroporation, sonication, surface engineering, and integration with biomaterials. Furthermore, this article discusses the prevailing challenges faced in engineered ABs, such as unsatisfactory isolation and purification yields, difficulties in differential separation of subtypes, the complexities in distinguishing successfully engineered ABs from their unmodified counterparts, the high heterogeneity among different ABs, and the yet‐to‐be‐completely elucidated vesicular contents. It is hoped that this manuscript will shed light on engineering strategies for ABs, thereby promoting their translational and clinical applications in the future.

## 2 OVERVIEW OF ABS

ABs are a specific type of EVs with sizes ranging from 1000‐5000 nm. As shown in Figure 1, ABs form in the terminal phase of cell apoptosis through three strictly regulated stages, namely: (a) Plasma membrane blebbing: Caspase‐3 activates p21‐activated kinase 2 (PAK2), Lim domain kinase 1 (LIMK1), and rho‐associated kinase 1 (ROCK1), leading to intracellular actomyosin contraction, generating compressive stress which increases intracellular hydrostatic pressure, causing the cytoplasmic fluid to bubble out in areas with weaker membrane attachment.^28^ (b) Apoptotic membrane protrusion formation: After membrane blebbing, some cells can continue to produce long apoptotic membrane protrusions, known as apoptopodia and beaded apoptopodia. Apoptopodia promotes the formation of ABs by separating vesicles.^29^ Beaded apoptopodia consists of several ABs and the fragmentation of a single beaded apoptopodia strand can lead to the release of 10–20 generally uniform single ABs.^30^ Many key molecular factors and processes are involved in regulating the formation of apoptotic membrane protrusions, including the negative regulation of the formation of apoptotic membrane protrusions by the pannexin 1 (PANX1) channel,^29^ the positive regulation of cytoskeletal rearrangement by the Plexin B2 (PlexB2) receptor,^31^, ^32^ and the maintenance of the structure of apoptotic membrane protrusions by F‐actin and microtubule tips.^33^ Inhibiting vesicle transport can also prevent the formation of ABs and beaded ABs without affecting cell apoptosis, membrane blebbing, and the activity level of PANX1.^30^ (c) ABs fragmentation: The final step of the disassembly process where apoptotic cells divide into discrete ABs has not been well‐studied. Some believe that the forces driving the fragmentation into ABs are extrinsic and intrinsic factors within the in vitro environment, such as shear forces generated by culture medium movement and physical interactions between apoptotic cells and neighboring cells.^17^ Notably, this highly regulated AB formation process will affect the contents of ABs. For instance, the DNA distribution index of ABs produced from Jurkat T cells treated with trovafloxacin by inhibiting PANX1 increases significantly, while trovafloxacin and GSK 269962 simultaneously inhibiting ROCK1 and PANX1 result in a significant decrease in the DNA and mitochondrial distribution index of ABs,^34^ indicating that the contents of ABs are closely related to the disassembly mechanism of apoptotic cells.

Upon the end of apoptosis, the “find‐me” and “eat‐me” signaling pathways are indispensable for the recruitment of phagocytic cells and subsequent clearance,^35^ preventing cellular content leakage and secondary necrosis (Figure 1).^36^ With the progression of apoptosis, apoptotic cells emit soluble mediators such as ATP, UTP, and CX3CL1/fractalkine, establishing a chemotactic gradient conducive to phagocytic migration, and characterizing the “find‐me” signal.^37^ The “eat‐me” signal is typified by the externalization of PS on the AB surface,^38^ facilitating phagocytic recognition and eventual efferocytosis. Complementarily, proteins such as calreticulin (CRT) and intercellular adhesion molecule‐3 (ICAM‐3) partake in this signaling cascade, further enhancing the specificity and efficiency of the “eat‐me” process.^39^, ^40^ Based on this “find‐eat” property, unengineered ABs can specifically target phagocytes such as macrophages in vivo, which cannot be achieved by other extracellular vesicles such as exosomes and microvesicles, and therefore ABs have their own unique advantages in immunomodulation and immunotherapy as well as in targeting cargoes to phagocytes, which will be discussed in more detail below.

ABs inherit organelles, nucleic acids (DNA, coding RNA, non‐coding RNA), lipids, proteins, and other apoptosis‐associated molecules from parent cells.^41^ Three databases, namely ExoCarta, EVpedia, and Vesiclepedia, have been established to catalog and analyze the biomolecules of ABs across different tissues and organs. Additionally, ABs can be identified through RNA profiling and proteomics. Biomarkers associated with ABs include PS,^17^ histones and DNA,^42^ caspase3,^43^, ^44^ caspase‐cleaved proteins like PANX1 and ROCK1, membrane‐associated protein V,^16^, ^45^ as well as cell‐type‐specific surface markers such as CRT, calnexin, and BiP/GRP78.^24^, ^46^ Moreover, numerous other potential biomarkers remain unidentified.

**FIGURE 2:**
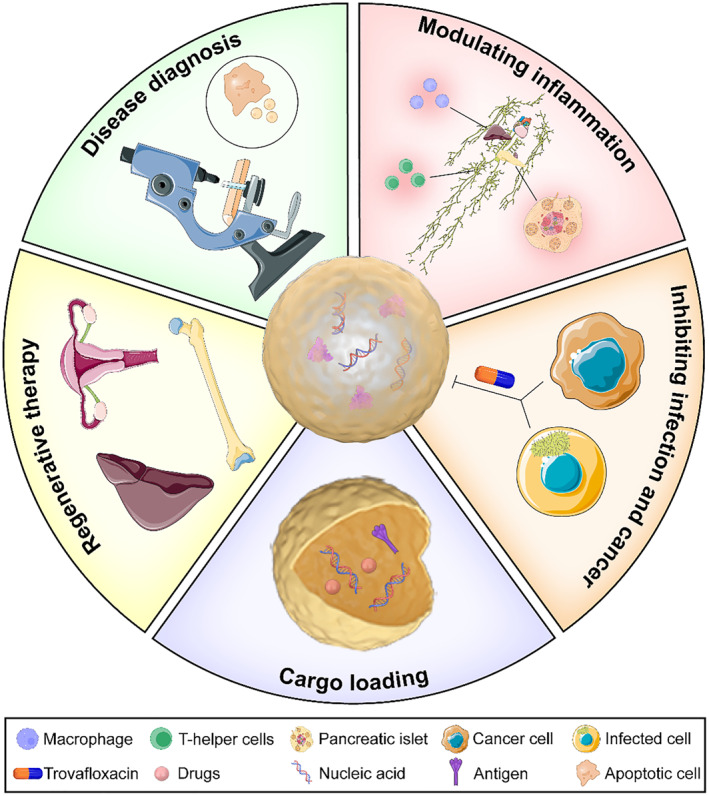
Current applications of ABs.

The biological functions of ABs are determined by their parent cells and influenced by the cellular state, growth conditions, and interactions of surrounding cells.^47^ The formation of ABs inherently mediates two critical roles: (a) assisting in apoptotic cell clearance, and (b) mediating intercellular communication by delivering biomolecules such as nucleic acids and proteins.^17^ Based on the two critical roles above, the applications of ABs and their corresponding mechanisms include: (a) Deficiencies in ABs are related to the clearance of apoptotic cells. Impaired release and defects in apoptotic cell clearance can lead to inflammation and autoimmunity,^48^ as observed in systemic lupus erythematosus, which triggers autoreactive T helper responses.^49^ Utilizing ABs can aid in alleviating inflammatory and autoimmune diseases, such as Type I diabetes,^50^ and modulating liver macrophage homeostasis to ameliorate Type II diabetes.^24^ (b) ABs derived from infected or cancerous cells may contain infectious agents or oncogenes. It's imperative to eliminate ABs from infected or cancerous cells to prevent the spread of viruses and the transmission of oncogenes.^51^, ^52^, ^53^ The quinolone antibiotic, trovafloxacin, is a novel PANX1 inhibitor currently in clinical trials that can pharmacologically block infections spreading by inhibiting the formation of ABs.^29^ (c) ABs can be engineered to carry drugs or biomolecules (like antigens from pathogens, DNA, RNA, and proteins) for vaccine preparation and targeted therapy. The large size of ABs gives it the advantage of higher cargo loading capacity compared to exosomes and microvesicles, making ABs a great potential in the field of cargo loading. In vaccine development, ABs have shown minimized off‐target effects and a more potent response.^54^, ^55^ Henry et al. reported that antigen‐presenting cells pre‐phagocytosed with ABs derived from murine colorectal cancer cells increased the survival rate of tumor‐bearing mice by 80%.^56^ It is important to note that although the contents of ABs are thought to be randomly distributed, they possess some properties arising from the apoptotic process, such as the autonomous production of hydrogen sulfide (H_2_S) to regulate the immune homeostasis of the body. If the purpose of the practical application is to utilize the inherent properties of ABs to achieve cell regeneration or immunotherapy, then there is no need to artificially alter the contents of ABs. However, if ABs are only used as carriers, and the actual therapeutic effect is the artificially loaded drugs in them, I believe that it is better to remove the original contents of ABs to prevent unknown side effects. (d) Stem cell‐derived ABs promote cell proliferation and differentiation, making them viable for regenerative therapies. Mesenchymal stem cell‐derived ABs achieved endometrial regeneration and fertility restoration when combined with hyaluronic acid (HA) hydrogel.^57^ Mature osteoclast‐derived ABs promote osteoblast survival and differentiation through activating NF‐κB (RANK) mediated PI3K/Akt/mTOR/Protein S6 kinase signaling.^58^ Hepatocyte‐derived ABs can induce JAK/stat‐mediated upregulation of the anti‐apoptotic protein Mcl‐1, promoting survival via stress‐related activation of the PI3K/Akt/NF‐κB cascade, thereby increasing cell survival rates.^59^ (e) Disease diagnosis. ABs have been used for diagnosing apoptotic colitis^60^ and histological grading of graft‐versus‐host disease (GVHD). In detail, histologically, GVHD is characterized by crypt apoptosis and crypt destruction. The original Lerner system focused mainly on crypt destruction without assessing the extent of apoptotic activity. Therefore, Ayesha et al. developed a novel histologic grading system for GVHD of the gastrointestinal tract that incorporates ABs from crypt biopsies into the grading criteria, allowing for a more precise diagnosis and assessment of prognosis.^61^ Recent reports suggested that a reduction in ABs derived from bone marrow osteoclasts is a characteristic feature of the pathological progression of osteoporosis.^62^ (Figure 2).

## 3 PREPARATIONS FOR OBTAINING ABS

Before ABs can be applied or engineered, what must be done first is to obtain sufficient quantities of ABs with high purity; however, as with other EVs, the induction, isolation, and purification of ABs vary very much from study to study. In the following section, the effects of the different methods of induction, isolation, and purification on the acquisition of ABs will be specifically discussed and their advantages and disadvantages will be evaluated.

### 3.1 Ways to induce ABs

Cells under normal culture conditions are unlikely to undergo apoptosis. Therefore, cells require specific stimuli to induce the production of ABs.^63^ Herein, the methods of AB induction in recent years are reviewed and summarized (Table 1).

**TABLE 1:** Modes of apoptosis induction and features of apoptosis patterns.

Processing method	Inducer	Features	Cell type	Reference
Chemical	Staurosporine	Simple and effective	Mesenchymal stem cells (MSCs)	24, 43, 44, 64, 65
			Adipose stem cells (ADSCs)	66, 67
			T Cells	68
			Osteoclasts	58, 62
			Fibroblasts	69
	Cisplatin	Cisplatin‐induced ABs inhibit HK‐2 cell growth and promote apoptosis	Proximal tubular HK‐2 cells	70
	α‐mangostin	Induces apoptosis while being loaded into ABs as an antioxidant and anti‐inflammatory agent	MSCs	71
	Alendronate	Specifically induces osteoclast apoptosis	Osteoclasts	72
	Metronidazole	Facilitates subsequent fluorescence labeling of nitro reductase imaging	Epithelial basal stem cells	73
	LPS	Simulates the immune response against bacterial pathogens	Macrophages	74
Physical	Ultraviolet rays	Cause DNA damage and programmed death	Macrophages	75
			Thymocytes and Jurkat cells	76
			Neutrophils	77
			MSCs	57
	Serum starvation	Promotes apoptosis by affecting the cell cycle	Endothelial cells	78, 79, 80
			Cardiomyocytes and Fibroblasts	81
			Osteoclasts	82
	High hydrostatic pressure	High efficiency and no exposure to chemical reagents	MSCs	83
Complex methods	Cisplatin Ultraviolet rays	Explore the metabolic differences of ABs in different apoptosis modes	Proximal tubular HK‐2 cells	84
	Tamoxifen 5‐Fluorouracil Cisplatin	Trigger different strengths of immune responses	Lewis lung carcinoma (LLC1) cells	85
	HIV + Acetaldehyde	Mimic a special disease model	Huh7.5‐CYP2E1 (RLW) cells	86

The most common stimulus for ABs is staurosporine, a Protein Kinase C inhibitor that induces cell apoptosis by activating caspase‐3 and inhibiting the AKT/MAPK pathway.^87^, ^88^ Serum starvation and ultraviolet irradiation are also common methods of inducing apoptosis. Other methods, including chemotherapeutic and cell‐specific apoptosis‐inducing drugs, have also been utilized and elucidated in specific studies. Le et al. suggested using 50 MPa of high hydrostatic pressure for 36 h to induce apoptosis in vitro.^83^ The advantage of this method is that the cells are not exposed to any chemical agents, and the efficiency of inducing apoptosis and ABs formation is comparable to that of 0.5 μM staurosporine‐induced MSCs apoptosis. It should be emphasized that the biological properties of ABs produced by different apoptotic inductions may not necessarily be the same, especially in immune responses. For instance, Sachdeva R et al. found that ABs immunized by tamoxifen in lupus‐prone mice produce a higher humoral immune response than those by cisplatin, and both types of ABs have poorer immunogenicity in healthy mice.^85^ Chattergoon et al. found that ABs produced by Fas signaling, toxic molecules exposure, or heat stress have a higher potential to induce dendritic cell maturation and CD8+ T cell immune response than those produced by DNA damage‐induced apoptosis pathways.^89^ While different apoptotic induction methods have been proven to affect the properties of ABs, related research is still limited.

Furthermore, it is known that some drugs can be loaded into ABs when they are co‐incubated with ABs, so is it possible that these apoptosis‐inducing drugs are also inadvertently loaded into ABs and play a role subsequently. Further studies are also needed to elucidate the process of inducing the production of ABs, which is the only way to completely control ABs from the very beginning and to produce optimally engineered ABs.

### 3.2 Ways to isolate and purify ABs

**FIGURE 3:**
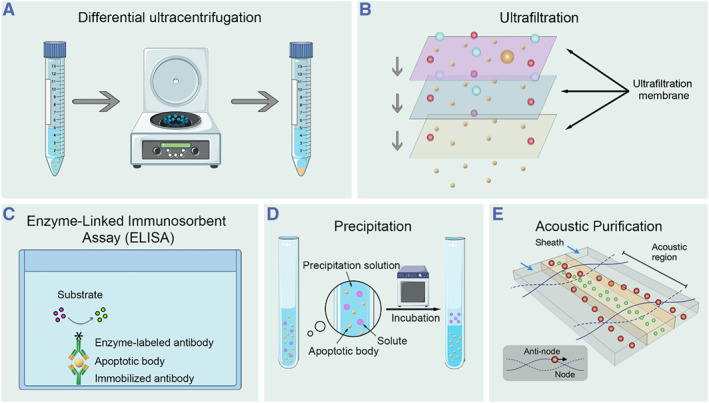
Common methods for separating and isolating ABs: (A) ultracentrifugation (represented by differential ultracentrifugation), (B) size‐based separation methods (represented by ultrafiltration), (C) affinity‐based separation methods (represented by ELISA), (D) precipitation, and (E) microfluidic‐based separation methods (represented by acoustic purification) (Reproduced with permission.^93^ Copyright 2015, American Chemical Society).

Before using ABs for any research or therapeutic applications, a critical challenge to address is the isolation and purification of ABs. As a specific type of EVs, the isolation and purification procedures for ABs should be standardized to eliminate contaminants such as peptides, proteins, lipids, and other types of EVs.^90^ With the advancement of molecular technology, the techniques for the isolation and purification of ABs are gradually being optimized.^91^, ^92^ Currently, the conventional methods for separating EVs include ultracentrifugation, size‐based separation methods, as well as some newer techniques such as Microfluidic‐based separation methods, affinity‐based separation methods, and precipitation (Figure 3). The advantages and limitations of each technique have been summarized, and they are suitable for application under different conditions (Table 2).

**TABLE 2:** Isolation and purification methods of EVs and their advantages and limitations.

Methods	Forms	Advantages	Limitations	References
Ultracentrifugation	Differential ultracentrifugation	Convenient Low preprocessing requirements	Time‐consuming Requires a large volume of samples	94, 95
	Density gradient centrifugation	Can separate EVs from proteins	Time‐consuming Low yield Requires larger sample volume	95, 96, 97
	Velocity sedimentation centrifugation	Can separate particles of the same density but different diameters Higher EVs recovery rate	Difficult to determine centrifugation duration	95, 98, 99
Size‐based separation	Ultrafiltration	Fast Convenient	Clogging effect High shear forces may damage vesicle integrity	100, 101
	Size exclusion chromatography (SEC) Gel permeation chromatography (GPC) Field Flow Fractionation (FFF)	High purity	Time‐consuming Requires specialized equipment	102, 103
Affinity‐based separation	Enzyme‐linked immunosorbent assay (ELISA)	High purity	Requires ultracentrifugation preprocessing for samples Expensive Low yield Limited available antibodies	95, 104, 105, 106
	Magnetic immunoprecipitation	Fast Can process large‐volume samples	Expensive	107
	Fluorescence‐activated cell sorting (FACS)	Fast Can process large‐volume samples	Expensive	108
Precipitation	Precipitation kits etc.	Fast Cheap Convenient	Poor selectivity and purity Must be combined with other methods	95, 104, 107, 109, 110
Microfluidic‐based separation	Acoustic purification Micro and nanofluidic chips combined with electrophoresis and electromagnetic technologies	Fast Cheap Efficient Suitable for small samples	Low production Lack of standardization	93, 95, 107, 111, 112

An ideal EV separation method should be relatively simple and inexpensive, without the need for complex or expensive equipment. It should be relatively fast, and allow for the separation from large samples.^107^, ^109^ Meanwhile, the strategy for AB purification should not be limited to a single method, and it can use a combination of techniques to achieve optimal efficiency, which requires further exploration.^113^, ^114^

## 4 ABS ENGINEERING

EVs exhibit lower immunogenicity and toxicity, higher biocompatibility^115^, ^116^, ^117^, ^118^ and circulation stability,^119^, ^120^ and the ability to cross physiological barriers and target specific tissues^121^, ^122^, ^123^ compared to synthetic nanocarriers and liposomes. Thus, they offer tremendous application potential in multiple fields. Additionally, ABs can reduce inflammation and target tissues precisely with their unique membrane proteins and “find‐eat” signals.^63^ However, the direct application of ABs can result in suboptimal therapeutic outcomes due to the following factors:

1. Highly heterogeneous content: The contents of ABs are thought to be randomly generated during the apoptotic phase, with varying sizes and contents among ABs, which is prone to random effect.
2. Uncontrollability: Uncontrollable original contents of ABs could lead to potential adverse side effects during treatment.
3. Rapid clearance rate: ABs are rapidly cleared by phagocytes when injected in vivo, making it difficult to maintain therapeutic concentrations. Additionally, High‐dose injections of ABs could lead to lung congestion and liver accumulation and cause cytotoxic effects.

Therefore, engineering modifications are necessary to achieve the desired outcomes with ABs. Engineering means can empty the contents of ABs and replace them with drugs or nanoparticles that clearly have a therapeutic effect, as well as achieve size homogenization of ABs, and also achieve locally targeted sustained release of ABs. In conclusion, engineering can be a very effective solution to the dilemma of ABs in application.

The engineering of ABs can be broadly categorized into three types: engineering of AB parent cells, direct engineering of ABs, and integration of ABs with biomaterials. These engineering strategies will be detailed in subsequent sections.

### 4.1 Engineering of ABs parent cells

The engineering modification strategy for AB parent cells is also known as the endogenous method. The principle is to overexpress the target nucleic acids, proteins, or drugs in the parent cells, thereby inducing the formation of ABs containing the target cargoes during apoptosis. Although manipulating cells is much easier than directly modifying ABs, there are still many unknown aspects in the process of ABs formation, especially the mechanisms of ABs membrane formation and the composition of contents, which makes the outcomes of the parent cell modifications unpredictable.^124^

#### 4.1.1 Metabolic modification

Metabolic modification is the simplest form of engineering modification for parent cells. By altering the conditions of cell culture, it is possible to regulate the synthetic and metabolic states of cells without the aid of genetic engineering, thereby indirectly manipulating the production and function of EVs. Conditions such as hypoxia, serum starvation, and drug stimulation can alter the production and functionality of EVs. For ABs, modulating the metabolic state of parent cells and selecting the mode of induced apoptosis are the most important. The previous section titled " 3. Ways to induce ABs " details how the mode of apoptosis impacts ABs. Modification of the metabolic state of parent cells will be discussed below.

Hypoxic modifications are commonly achieved through incubation at a low level of oxygen^125^ or with chemical hypoxia induced by agents such as dimethyloxallyl glycine or cobalt chloride.^126^ Research has found that hypoxia increases the release of small extracellular vesicles (sEVs) compared to normoxia.^125^ Additionally, the size of these vesicles also undergoes varying alterations depending on the degree of hypoxia.^127^ Furthermore, hypoxia‐induced extracellular vesicles (hypo‐evs) exhibit diverse enhanced characteristics in their effects on cells. For example, endothelial cells show increased survival rates and superior angiogenic capabilities after treatment with hypo‐evs.^128^ Hypo‐evs derived from MSCs have a protective effect against cell death in cardiomyocytes.^129^ The migration and invasive capabilities of cancer cells treated with hypo‐evs are enhanced.^130^ Immune cells post hypo‐evs treatment exhibit anti‐inflammatory effects.^131^, ^132^ In summary, hypo‐evs harbor significant potential in wound healing, myocardial protection, and immunotherapy. After treatment with hypoxia, a pathological environment, EVs not only become more abundant in terms of production but also enable the recipient cells to perform stronger cellular functions and greater survival capacity.

Serum starvation involves restricting cellular nutrition by reducing or eliminating serum from the culture medium, which can result in an increased production of EVs, and it also can influence the cell cycle, subsequently promoting cell apoptosis and the formation of ABs.^133^ Studies by Sun L. et al. have demonstrated that serum deprivation can stimulate human myeloma cells RPMI 8226, U266, and KM3, producing 2.5 times, 4.3 times, and 3.8 times more EVs, respectively,^134^ which offers insights into the bulk production of EVs. Wang et al. found that exosomes derived from starvation‐stressed THLE‐2 cells could enhance protective autophagy in hepatocellular carcinoma (HCC) cells and promote HCC progression.^135^ It is crucial to note that the extent of serum starvation impacts the induction of apoptosis and the formation of ABs. If the serum level is not low enough to induce apoptosis, it can cause the cells to activate compensatory autophagy. This process helps the cells to maintain their energy levels and prevents AB formation. On the other hand, if the cells' ATP levels are completely depleted, it can trigger apoptosis, but this does not result in AB production either. The mechanism of how the duration and extent of serum starvation treatment affects apoptosis and the number and nature of ABs requires further experimental elucidation.

Drug stimulation is achieved by incorporating stimulatory drugs such as LPS, TNF‐α, IFN‐γ, BMP‐2, and melatonin to mimic inflammatory or other environments, enhancing the anti‐inflammatory and regenerative abilities of EVs.^136^, ^137^, ^138^, ^139^ (Table 3).

**TABLE 3:** Ways of metabolic modifications and corresponding effects.

Metabolic modification	Effects on EV release	Effects of EVs on recipient cells	References
Hypoxia	Increase production Size change Change in contents	Enhance survivability Enhance angiogenic capabilities Enhance anti‐inflammatory effects	125, 126, 127, 128, 129, 130, 131, 132
Serum starvation	Increase production Produce ABs Change in contents	Enhance autophagy and survivability	134, 135
Drug stimulation (LPS, TNF‐α, IFN‐γ, BMP‐2, and melatonin)	Undetermined	Enhance anti‐inflammatory and regenerative abilities	136, 137, 138, 139

Many other metabolic modification methods for parent cells exist, such as electrical stimulation, electromagnetic waves, ultrasound, shear stress, alcohol, pH levels, and temperature. These methods have been proven to enhance the yield of EVs. However, the results of the modifications are closely related to the methods and degree of modification. Excessive modification can even lead to opposite results.^125^ Moreover, most of the current studies focus only on EVs as a whole or on exosomes and microvesicles. Few studies have isolated ABs to investigate changes through metabolic modification. Although ABs serve as one type of EVs, their production and functional variations can refer to those of exosomes and microvesicles. However, there still exist differences between them and other EVs. Zhu et al. found that early EVs after LPS stimulation are mainly ABs rather than exosomes or microvesicles.^74^ Mary C. Patton et al. observed a significant increase in sEVs of pancreatic cancer cells following hypoxia induction, while minimal induction was observed in medium and large EVs, such as ABs.^127^ Many mechanisms by which metabolic changes in parent cells affect ABs are not clear. Whether it alters the characteristics and other functions of ABs is also unknown.

#### 4.1.2 Direct manipulation of parent cell contents

Direct manipulation of parent cell contents is typically employed for loading nucleic acids or drugs into ABs, and the most prevalent methods in ABs are transfection and co‐incubation.

##### Transfection

**FIGURE 4:**
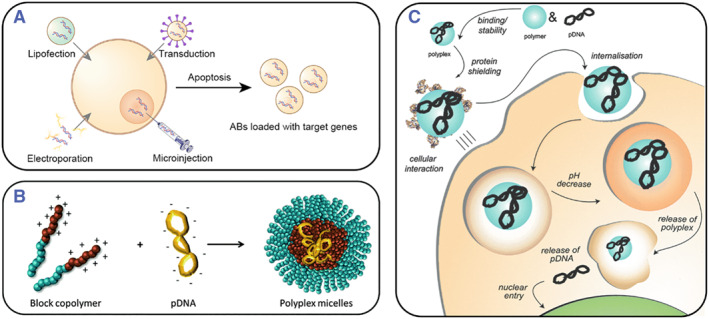
Schematic diagram of parent cell transfection. (A) The principle of parent cell transfection involves introducing the target nucleic acids into parent cells by various methods and then inducing apoptosis to generate ABs containing the target nucleic acids. (B) Cationic polymers can compress DNA structures and form stable complexes to protect DNA. (C) After cationic polymers encapsulate DNA, they enter the cell. Subsequently, the intracellular pH decreases, resulting in the dissociation of the polymer and consequent release of DNA inside the cell. (Reproduced with permission.^142^ Copyright 2015, The Royal Society of Chemistry).

Transfection is a process of introducing foreign nucleic acids into cells to generate transgenic cells. The introduced genetic material (DNA and RNA) or proteins exist stably or transiently in the cells, enhancing or inhibiting specific gene expression in the cells to produce recombinant proteins for various therapeutic purposes.^140^, ^141^ Transfection primarily includes physical, chemical, and biological methods (Figure 4A). Each method must be considered according to cell type and purpose. The ideal transfection method should be efficient, non‐cytotoxic, convenient, and reproducible.^143^ Shin Sasaki et al. bio‐transfected parent cells with vectors co‐expressing influenza virus hemagglutinin (HA) or nucleoprotein (NP) genes and mutant caspase genes, obtaining antigen‐loaded ABs. These ABs significantly increased T‐cell responses, offering a new approach to immunotherapy.^144^ Zhu et al. used chemical agents to transfect macrophages with miR‐221 and miR‐222 inhibitors and then studied the change of macrophage‐derived ABs miRNA after LPS stimulation.^74^ Zheng et al. utilized small interfering RNA (siRNA) to transfect MSCs to eliminate CRT expression on the surface of ABs, thereby studying the phagocytosis and anti‐inflammatory functions mediated by CRT in ABs.^24^

Meanwhile, the rational utilization of cationic polymers can enhance the efficiency of transfection. As shown in Figure 4B, cationic polymers bind with the negative charge of DNA or RNA, compacting the structure and forming stable complexes that encapsulate the target genes, thus protecting them from nuclease degradation. As shown in Figure 4C, after cationic polymers enter cells, the pH decreases and the polymers release nucleic acids, enabling gene expression.^142^ Wang et al. developed a targeted drug delivery strategy using ABs to reach brain cells. First, anti‐tnf‐α antisense oligonucleotides (ASO) were combined with cationic konjac glucomannan (cKGM), and then brain microvascular endothelial cells (BMECs) expressing mannose receptors (MRs) were chemically transfected with cKGM. Eventually, ultraviolet light and H2O2 induced apoptosis to form cKGM/ASO complex‐containing ABs (CABs). These CABs can cross the blood‐brain barrier and are ultimately taken up by microglia in the brain, exhibiting extremely high brain delivery efficiency.^145^ It has been proven in a mouse model of Parkinson's disease that PD symptoms can be significantly improved through the anti‐inflammatory action of CABs.

Similarly, cations such as calcium ions and magnesium ions can also be utilized to enhance transfection. For example, Maloverjan et al. found a way to improve the transfection efficiency of nanoparticles made with cell‐penetrating peptides (CPP) and splice‐correcting oligonucleotides (SCO) by incorporating calcium and magnesium ions into the nanoparticles. This resulted in increased synthesis of functional proteins and higher therapeutic potential without having to increase the peptide concentration.^146^ Wilson et al. showed that poly *β*‐amino esters (PBAEs) are excellent transfection agents. Moreover, they discovered that using a buffer solution of divalent cations such as magnesium or calcium acetate with a pH of 5.0 as the transfection buffer instead of monovalent cations led to a several‐fold increase in transfection efficiency.^147^ In summary, the auxiliary application of cations enhances the robustness of applications in bioengineering and biotechnology.

##### Co‐incubation

**FIGURE 5:**
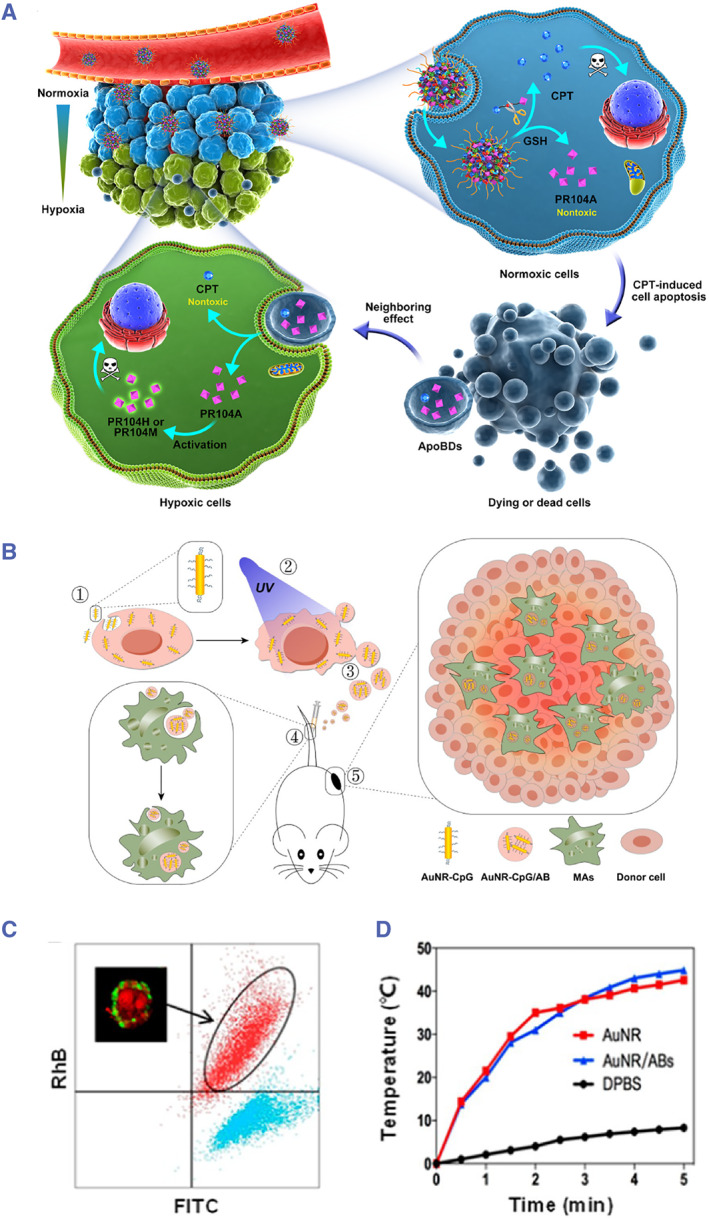
(A) Schematic diagram of CPT and PR104 A enhancing drug penetration and whole tumor destruction through ApoBD‐mediated neighboring effect. (Reproduced with permission.^148^ Copyright 2021, The Authors, published by American Association for the Advancement of Science). (B) Schematics of the AuNR‐CpG/AB system for enhanced tumor photothermal immunotherapy. Donor cells were first fed with CpG‐modified AuNR and then irradiated with UV light to generate AB‐encapsulated AuNR‐CpG. After intravenous injection into tumor‐bearing C57BL/6 mice, AuNR‐CpG/AB could be rapidly phagocytized by circulating monocytes/macrophages (MAs). With the natural tumor‐homing tendency of MAs, AuNR‐CpG/AB could be efficiently delivered to the inner region of a tumor. Then, NIR laser irradiation was applied to ablate the tumor through the photothermal effect of AuNR. (C) Flow cytometric analysis of AuNR/ABs (red) and ABs (blue). AuNR was coated with RhB‐embedded silica. Both AuNR/ABs and ABs were stained with Annexin V‐FITC. Inset: corresponding CLSM image of AuNR/ABs. (D) Temperature–time curves of the medium with AuNR (red), AuNR/AB (blue), and DPBS (black) with NIR laser irradiation (808 nm, 2 W/cm2). The corresponding concentration of AuNR in each sample was fixed at 2.1 μg. (Reproduced with permission.^149^ Copyright 2020, American Chemical Society).

Co‐incubation involves directly culturing parent cells with transgenes or drugs intended for loading without the mediation of external factors. Apoptosis is then induced, producing ABs with the desired cargo. Zhao et al. directly co‐incubated tumor cells with camptothecin (CPT) and the hypoxia‐activated prodrug PR104 A made up of prodrug nanoparticles. While CPT exerted its cytotoxicity killing the normoxic external tumor cells, it also entered the ABs formed by apoptotic tumor cells, leading to the formation of ABs co‐loaded with CPT and PR104 A. When these ABs penetrated deep into the hypoxic center of the tumor, though the toxicity of CPT was reduced, PR104 A was activated and its strong toxicity led to the death of deep‐seated tumor cells (Figure 5A).^148^ This study skillfully utilized two drugs that effectively exert their toxicity under two oxygen conditions. With the aid of ABs, they achieved a high‐efficiency killing capability from the external part of the tumor to its deep regions.

Bao et al. developed a lipid‐coated nanoparticle loaded with 2′, 3′‐cyclic guanosine monophosphate‐adenosine monophosphate (cGAMP). The internal core of this nanoparticle consists of Fe(II) ions and cGAMP molecules. After co‐incubating the lipid‐coated nanoparticles with tumor cells, they enter the cytosolic gel, releasing free Fe(II) ions and cGAMP molecules. These subsequently initiate the Fenton reaction and the STING pathway working in tandem to promote tumor cell apoptosis.^150^ This results in the generation of ABs carrying exogenous cGAMP. These ABs are readily phagocytosed by APCs, enhancing the body's adaptive immunity against tumors.

Zheng et al. incubated tumor cells with gold‐silver nanorods (AuNR) modified with cytosine‐phosphate‐guanine (CpG), and upon ultraviolet irradiation, they induced the formation of ABs loaded with the nanomedicine (AuNR‐CpG/ABs). These ABs can be specifically phagocytosed by inflammatory monocytes, leveraging their natural tumor‐homing propensity to actively infiltrate the tumor core. Through the photothermal effect of the nanorods and the immune stimulation of CpG, they managed to ablate the tumor and prevent metastatic relapses (Figure 5B–D).^149^ This study ingeniously utilized the homing characteristic of tumor cell ABs for targeted tumor therapy, combining the physical and biological effects of the nanorods loaded within the ABs to eliminate tumor cells. Although the photothermal effect of the nanorods in this study requires near‐infrared light illumination, which has limited penetration in human tissues making it potentially challenging for in vivo applications, and the loading efficiency of AuNR in the experiment was relatively low, it still offers insights into using ABs for targeted tumor therapy.

Although the types of target cargoes for transfection and co‐incubation differ, they are essentially similar, both utilizing different methods to load the desired cargo into living cells. Nucleic acids and proteins require carriers to enter cells, whereas drugs and lipid‐coated nanoparticles require membrane fusion or active cellular uptake to enter cells. Efficient loading of cargo depends on various factors such as carrier type, biophysical properties of the cargo, and physiological state of the target cells. It is important to consider rational carrier design, pre‐treatment of cargo, and use of auxiliary reagents before proceeding with loading operations. All of these will help to achieve the highest and most stable loading efficiency.

#### 4.1.3 Surface engineering of parent cells

**FIGURE 6:**
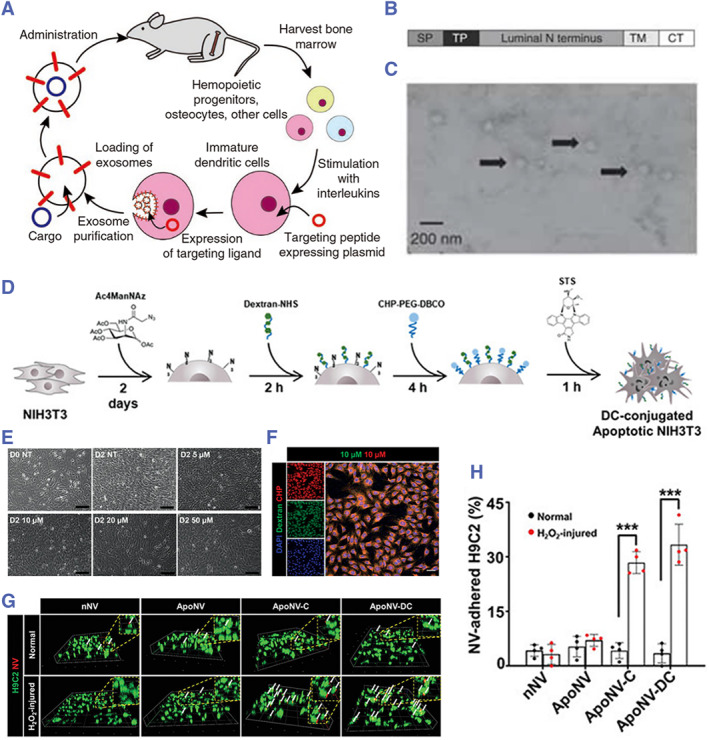
(A) In parent cells, target proteins are coupled to known exosomal conservative non‐specific membrane proteins, producing exosomes carrying target proteins. (B) Schematic representation of the modified Lamp2b protein. Targeting peptides are fused to the extra‐exosomal N terminus of murine Lamp2b. (SP, signal peptide; TP, targeting peptide; TM, transmembrane domain; CT, C terminus.) (C) Electron micrograph of phosphotungstic acid stained RVG exosomes. (Reproduced with permission.^151^ Copyright 2011, Springer Nature). (D) Directly modifying the surface of parent cells, followed by apoptosis induction, directly produces surface‐engineered ApoNVs. (E) Bright‐field images of NIH3T3 cells after the Ac4ManNAz treatment at various concentrations. Scale bars = 100 μm. (F) Confocal microscopic images showing the conjugation of NIH3T3 cells with dextran (green) and CHP (red). Blue indicates DAPI. Scale bars = 50 μm. (G) 3D fluorescent images and (H) the quantification data showing the attachment of NVs on the membrane of H9C2 cells 3 h after the NV treatment. Scale bars = 100 μm. White arrows indicate NVs (red) on the H9C2 cell membrane (green). (Reproduced with permission.^69^ Copyright 2023, The Authors, published by John Wiley and Sons).

The indirect AB surface engineering mainly involves two strategies. One method involves chemically modifying or genetically manipulating target proteins to couple them with known EV membrane proteins. This specific membrane protein then loads the target protein onto the membrane surface of EVs. This method has been reported in exosomes and microvesicles. Matthew Wood and colleagues engineered dendritic cells to produce neuro‐targeted exosomes by fusing Lamp2b (an exosome membrane protein) with the RVG peptide (Figure 6A–C).^151^ In exosomes, conservative non‐specific membrane proteins available for coupling include Lamp2b, CD9, CD63, and CD81.^152^ This surface engineering strategy has not yet been applied to ABs, likely due to the absence of identified conservative non‐specific membrane proteins in ABs suitable for coupling. Another strategy for surface engineering involves the direct modification of the parent cell membrane using click chemistry techniques. Lee et al. conducted an experiment in which they initially incubated NIH3T3 cells with Cy3‐glycogen‐NHS. They then treated the cells with Ac4ManNAz for 2 days, which helped to express azide groups on the cell membrane. Finally, the cells were incubated with Cy3‐CHP‐PEG‐DBCO, which facilitated the conjugation of glycogen and ischemic heart‐homing peptide (CHP) to the NIH3T3 cell membrane (Figure 6D–F). The dextran and/or CHP‐conjugated NIH3T3 cells were then treated with staurosporine and extruded through 10, 5, and 0.4 μm‐sized porous polycarbonate filters to produce apoptotic bodies‐mimetic nanovesicles (ApoNVs). The produced ApoNVs had enhanced targeting capabilities due to CHP and were used in the treatment of cardiac ischemia‐reperfusion injury, potentially preventing cardiac remodeling and mitigating the deterioration of cardiac function (Figure 6G,H).^69^ This surface engineering strategy combined the biogenic characteristics of ABs membrane blebbing, allowing for direct modifications on the surface of progenitor cell membranes, simplifying the process of engineering the surface of ABs.

In summary, there are three approaches to modify ABs: metabolic modification, content alteration, and surface engineering of parent cells. These approaches modify ABs indirectly by altering different parts or physiological processes of the cell. Although these three engineering methods differ in perspective, they are not contradictory and can be used in combination to achieve optimal results. The advantage of parent cell engineering is that cell manipulation is simpler than vesicle manipulation. However, the drawback is that the biogenesis mechanism of ABs is not yet fully understood. Moreover, the membrane and content of ABs have some randomness. The process of obtaining target ABs from engineering modified parent cells is not fully controllable, and the modification results are unpredictable, all of which are issues that need consideration and resolution.

### 4.2 Direct engineering modification of ABs

Modifying ABs is more direct and controllable than modifying parent cells as it bypasses the unpredictable AB biogenesis process. Similarly, direct engineering strategies for ABs can be divided into two categories: direct manipulation of AB content and surface engineering. It's essential to ensure the structural stability of ABs during their direct engineering modification. Any damage to key membrane proteins or membrane rupture could be counterproductive.

#### 4.2.1 Direct manipulation of ABs content

**FIGURE 7:**
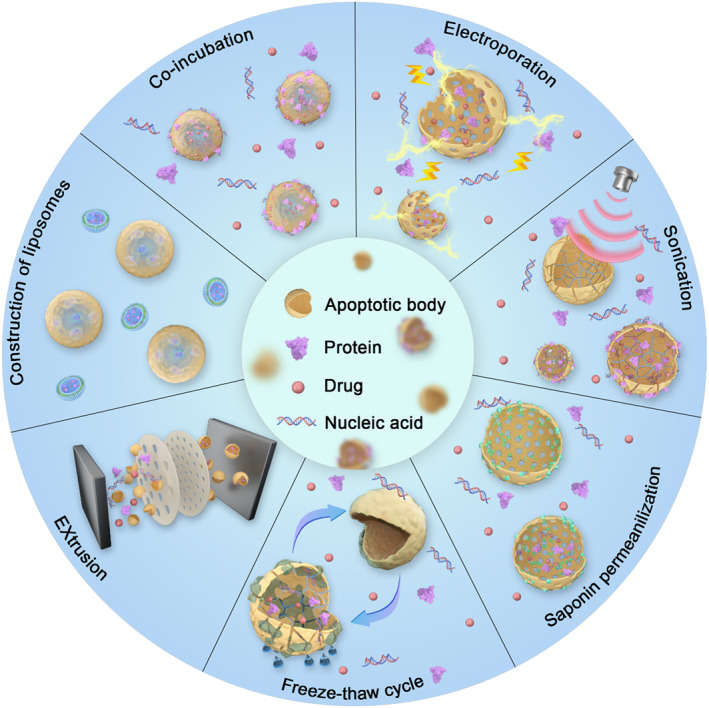
Direct Manipulation of AB Content (co‐incubation, electroporation, sonication, saponin permeation, freeze‐thaw cycle, extrusion, construction of liposomes).

The first step in modifying ABs is to break through their bilipidic membrane. Hydrophobic molecules can be passively integrated by co‐incubation due to their nature, allowing them to traverse lipid bilayers. In contrast, hydrophilic molecules require techniques such as electroporation, sonication, saponin permeabilization, freeze‐thaw cycles, extrusion, and construction of liposomes to be loaded into ABs (Figure 7).^153^, ^154^, ^155^, ^156^

You et al. encapsulated a hydrophobic drug, *α*‐mangostin (α‐M), into ABs through co‐incubation. The hydrophobic interactions occurring between the lipid membranes facilitated this. ABs were then used to modulate inflammation and repair tissue, which improved the prognosis of ischemic stroke.^71^ While this direct co‐incubation method is operationally simple, it has stringent requirements for the encapsulated cargo. If the intended cargo is hydrophilic, other modification strategies would be necessary.

In practical applications, it is common to integrate several engineering techniques to attain the best therapeutic outcomes. Rajendran J. C. Bose et al. first ultrasonicated purified cancer cell ABs to break them into smaller fragments. Following this, a freeze‐thaw cycle in the presence of vancomycin was employed to introduce vancomycin into the ABs. They then passed the vesicle suspension of ABs through polycarbonate filters with decreasing pore sizes (ranging from 0.4 to 0.2 μm), which resulted in the reconstruction of ABs with a consistent size between 100 and 150 nm.^82^ This method not only ensures consistent vesicle size but also achieves the highest encapsulation of vancomycin. These engineered vesicles can specifically target macrophages and cancer cells, effectively killing intracellular *Staphylococcus aureus* infections.

**FIGURE 8:**
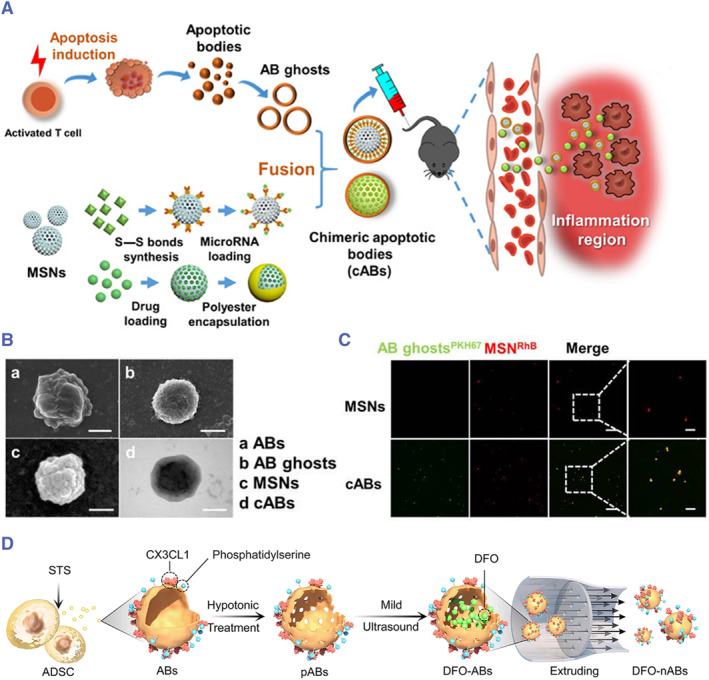
Schematic illustration of the ABs content emptying, membrane retrieval, and subsequent reloading. (A) Removal of ABs content through hypotonic treatment and ultrasonication, extraction of the membrane followed by fusion with silica nanoparticles carrying miR‐21 or curcumin, resulting in the formation of chimeric ABs. (B) Representative SEM images of ABs. Scale bars, 1 μm (a), 500 nm (b), and 100 nm (c, d). (C) Representative fluorescence images show the colocalization of MSNs and AB ghosts after manipulation of cABs. Scale bars, 20 (left) and 5 μm (right). (Reproduced with permission.^68^ Copyright 2020, The Authors, published by American Association for the Advancement of Science). (D) DFO‐nABs were obtained by mixing drugs along with sonication after hypotonic treatment. (Reproduced with permission.^66^ Copyright 2023, Elsevier).

G. Dou et al. employed a combination of hypotonic lysis, ultrasonication, and differential centrifugation to empty the intracellular content of ABs (Figure 8A). This process effectively removed residual components of ABs while preserving their membrane and innate targeting properties. The resulting product was referred to as “AB ghosts” (Figure 8B). Subsequently, they introduced mesoporous silica nanoparticles (MSN) containing anti‐inflammatory agents, such as microRNA‐21 (miR‐21) or curcumin (Cur), into the ABs through membrane fusion (Figure 8C). This creation, termed chimeric ABs, has shown promise in improving the conditions of skin inflammation and inflammatory bowel diseases, promoting overall regeneration.^68^ This strategy of only retaining the membrane structure of ABs, while eliminating the content, not only mitigates potential side effects from uncharacterized components inside ABs but also harnesses the robust targeting potential inherent to ABs, offering a fresh perspective on ABs applications.

Similarly, Bao et al. isolated the neutrophil apoptotic body membrane (NABM) from ABs by hypotonic and ultrasonic treatment. They then co‐extruded a mixture of NABM and MSN through a 200 nm polycarbonate membrane for at least 10 cycles, obtaining hexyl 5‐aminolevulinate hydrochloride (HAL)‐loaded engineered neutrophil ABs, which played anti‐inflammatory roles and enhanced cardiac function during myocardial infarction repair.^157^ This approach mimics the physiological process of abundant neutrophil apoptosis post‐myocardial infarction, leveraging engineered ABs for anti‐inflammatory regeneration, thereby aiding recovery after a heart attack.

Inspired by this model, Qian et al. prepared deferoxamine‐functionalized nano‐adipocyte ABs (DFO‐nABs) through the process of hypotonic treatment, mild ultrasonication, drug mixing, and extrusion (Figure 8D).^66^ This refined method achieves a high encapsulation efficiency for water‐soluble drug molecules and can target endothelial cells, upregulating the expression of vascular endothelial growth factor (VEGF), thus promoting angiogenesis and wound healing.

The method of removing cellular contents can be summarized as initially utilizing hypotonic and ultrasonic treatments to clear the contents of ABs. Subsequently, after washing, differential centrifugation was used to collect and purify the concentrated apoptotic membranes. The apoptotic membrane suspension is extruded through a polycarbonate membrane to form nano‐apoptotic vesicles and finally mixed and extruded with drugs to generate the ultimate engineered ABs. Qian et al. innovated and simplified the procedure by directly mixing drugs during ultrasonication.

#### 4.2.2 Surface engineering of ABs

**FIGURE 9:**
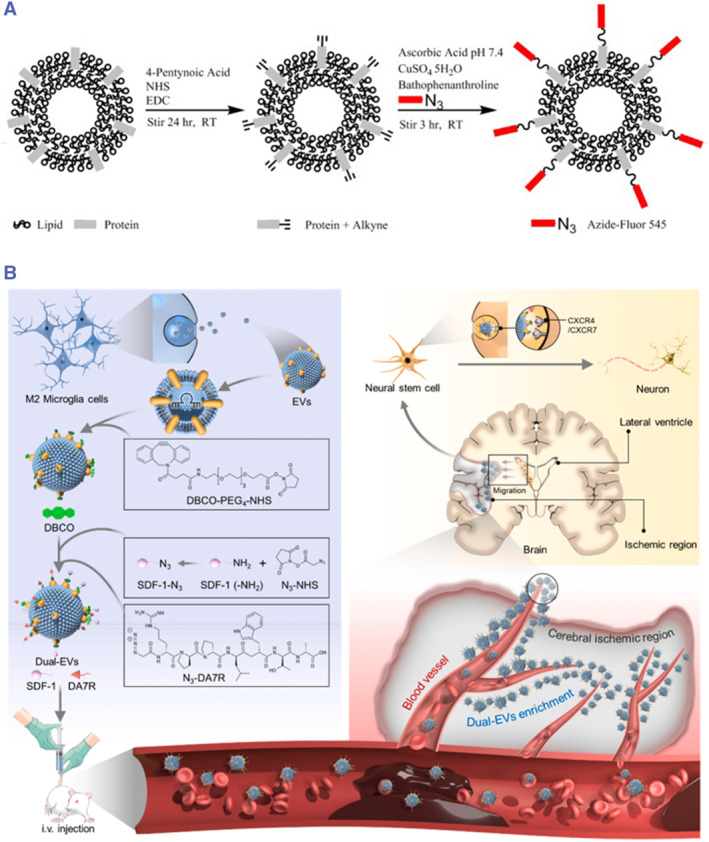
Surface engineering using click chemistry techniques. (A) Copper‐catalyzed azide‐alkyne cycloaddition links the fluorescent molecule azide‐fluor 545 dye to the surface of exosomes. (Reproduced with permission.^159^ Copyright 2014, American Chemical Society). (B) Copper‐free click chemistry allows the coupling of injured vascular targeting peptide (DA7R) and the stem cell recruiting factor (SDF‐1) to the surface of EVs, recruiting NSCs and promoting differentiation at central nervous system injury sites. (Reproduced with permission.^160^ Copyright 2023, Elsevier).

The abundance of amino groups and alkyl chains on the AB membrane makes surface engineering feasible. Although there are no relevant reports regarding ABs, such applications have been observed in other types of EVs. Wu et al. drew inspiration from the externalized PS structure of ABs. They prepared apoptotic body‐mimicking liposomes (AP‐Lipo) with engineered PS linked to their surface by blending liposomes with PS dissolved in saline, followed by homogenization using an ultrasonic probe in an ice bath. These were targeted towards macrophages to stabilize atherosclerotic plaques.^158^ T. Smyth et al. employed copper‐catalyzed azide‐alkyne cycloaddition (CuAAC) to conjugate the fluorescent molecule azide‐fluor 545 dyes to the exosome surface after using alkyne‐activated amines.^159^ This click chemistry is characterized by its rapid reaction time, high specificity, and excellent compatibility in aqueous buffers. After successfully conjugating the ligands to the exosome surface, they achieved staining and localization of exosomes. It was demonstrated that this click chemistry approach did not affect the size of the exosomes or their binding affinity to target cells (Figure 9A). The advantage of this surface modification method lies in its ability to directly couple or modify membrane proteins of ABs, further influencing cellular signaling to control the targeting specificity of ABs. However, a drawback is that it might induce peroxidation of unsaturated phospholipids in the ABs membrane by copper ions, leading to changes in permeability and compromised integrity, making it prone to leakage.^161^ Furthermore, copper ions have potential cytotoxic effects on cells. Based on this limitation, Carolyn Bertozzi developed a copper‐free click chemistry technique. Subsequently, many researchers utilized copper‐free click chemistry to engineer EVs. For instance, Xu et al. employed this novel method to fluorescently label exosomes for pancreatic cancer treatment, achieving intracellular tracking and quantification of cell uptake.^162^ Ruan et al. applied copper‐free click chemistry to modify the surface of EVs secreted by M2 microglia with injured vascular targeting peptide (DA7R) and the stem cell recruiting factor (SDF‐1) to recruit neural stem cells (NSCs).^160^ This promoted the differentiation of NSCs at central nervous system injury sites, offering new insights into the regeneration of neurons after CNS injuries using EVs (Figure 9B). This novel copper‐free click chemistry technique has the potential for surface engineering modifications of ABs.

### 4.3 Integration of ABs with biomaterials

The functionality and targetability of ABs can be improved through drug loading or surface engineering. However, when applied in vivo, rapid clearance can be an issue. Patients need to be injected with ABs several times in a short period, which not only causes great pain but also causes liver and lung accumulation and cytotoxic effects, both of which are unfeasible. Integration with biomaterials is essential to ensure the prolonged and sustained release of ABs. Moreover, the properties of biomaterials can be tailored according to the structural characteristics of the injection site, offering flexible adjustments of physicochemical properties to meet specific needs.^163^, ^164^ The following section explores the integration of ABs with biomaterials in a variety of diseases as well as characterizing the selection of specific biomaterials for different disease models.

**FIGURE 10:**
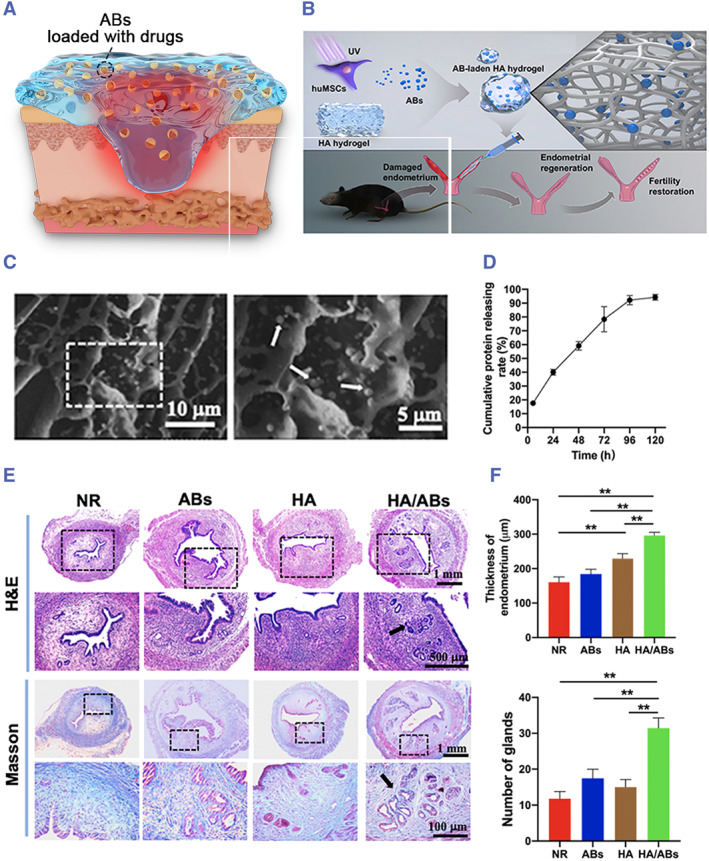
(A) Hydrogels can enable the sustained release of ABs at the wound site. (B) Schematic illustration of the design and application of the AB‐laden HA hydrogel. The hydrogel consisted of ABs produced from apoptotic MSCs and was administered into the uterine cavity via in situ injection to promote endometrial regeneration and restore fertility. (C) Scanning electron microscopy (SEM) images of the AB‐laden HA hydrogel. (D) The cumulative protein‐releasing profile of ABs from the AB‐laden HA hydrogel in the presence of hyaluronidase. (E) Hematoxylin and eosin (H, E) staining and Masson's trichrome staining images of uteri under different treatments for 12 d. (F) Quantification of endometrial thickness and endometrial gland under different treatments for 12 d. (*n* = 11, *p* < 0.01). (Reproduced with permission.^57^ Copyright 2022, The Authors, published by Elsevier).

In various disease models, the most frequent application of ABs combined with biomaterials is in the skin wound healing model (Figure 10A). For wound healing models, dressings must possess effective hemostasis, antibacterial properties, exudate absorption, breathability, water permeability, and tissue adhesion capabilities. Hydrogels aptly meet the physiobiological requirements for wound healing, and their combination with ABs has seen some applications. Liu et al. embedded MSC‐ABs into PF‐127 hydrogel and observed a faster skin regeneration rate compared to the MSC‐ABs‐alone group and the hydrogel group.^43^ Xin et al. combined mesenchymal stem cell‐derived ABs with hyaluronic acid (HA) hydrogel (Figure 10B). The HA hydrogel enhanced the retention of ABs and promoted their sustained release (Figure 10C,D). In mouse models of acute endometrial injury, in‐situ injection of ABs‐loaded HA hydrogel effectively reduced fibrosis and promoted endometrial regeneration.^57^ (Figure 10E,F) Sheng et al. developed an AB carrier based on imaging guidance. They combined engineered ABs with CD47 antibodies‐loaded hydrogel to enhance innate and adaptive immunity, regulate macrophage polarization, and modulate the immunosuppressive microenvironment, further boosting anti‐tumor immunotherapy. This combination showed promising therapeutic effects in a 4T1 breast tumor‐bearing mouse model.^75^

In other disease models, there is significant potential in combining ABs with biomaterials. In models of cardiovascular disease, combining ABs with methacrylate hyaluronic acid (HAMA) hydrogel can fill local defects caused by myocardial infarction. Alternatively, pairing heparin‐loaded ABs with vascular stents can enhance the long‐term patency of the stent. In models of osteochondral disease, a combination of ABs and poly (lactic acid hydroxyglycolic acid) copolymer (PLGA)‐‐based rigid organic scaffolds may promote bone formation. On the other hand, hydrogels such as gelatin and hyaluronic acid are better suited for treating cartilage and intervertebral disc diseases due to their compression‐bearing active structures. In neural regeneration models, it might be beneficial to combine ABs with hydrogels that modulate the microenvironment, nanotubes that guide neural growth, and conductive fibers to direct accurate neuronal growth and neural docking.^165^ Exosomes and microvesicles have been used to apply many of these strategies, providing insight into the engineering and use of ABs.^124^

## 5 CONCLUSION

As stated in this article, ABs are not merely the “garbage bags” after cell death, but rather mediums that play crucial regulatory roles in both the physiological and pathological processes of cells. ABs have the functions of regulating inflammation, loading cargo, and promoting regeneration. Some ABs can even penetrate physiological barriers to reach locations that other nanocarriers cannot access. ABs possess advantages like low immunogenicity, high targeting, and high drug‐loading capacity. However, they also have drawbacks including unclear mechanisms, uncertain biosafety, and rapid clearance in the body. Thus, there is a need for comprehensive engineering of ABs, from induction and separation to specific modification and integration, to achieve the desired therapeutic effects.

As this review comprehensively elucidates, engineering perspectives on ABs can be divided into three categories: indirect modification through parent cells, direct modification, and integration with biomaterials. The following conclusions and trends for future developments can be noted:

1. The advantage of modifying the parent cell lies in the simpler cell modification, but since the mechanism of how parent cell modification affects ABs is not yet fully understood, the derived ABs might not be entirely controllable. The need for further mechanistic elucidation of the biogenesis of ABs is essential.
2. Directly modifying ABs bypasses this “black box” process, but the challenge lies in the intricate vesicle manipulation and potential membrane disruption. Innovative studies have employed hypotonic and ultrasonic treatment to extract AB contents leaving pure AB membranes for cargo loading. This method has the advantage of preserving the targeting and biocompatibility of the AB membrane, ensuring a higher cargo loading rate, and reducing unpredictable side effects. Recent studies have also shown optimized operational procedures for this kind of use. Finding the most suitable engineering approaches and combinations is an important future research direction.
3. Current research on ABs combined with biomaterials is limited, mainly applied in wound healing models to achieve sustained release, addressing the issue of rapid clearance in circulation. Further design ideas for integrating ABs with biomaterials are also briefly described in this review. In the future, on the one hand, the combination of different biomaterials and ABs can be tried to treat diseases or realize regenerative therapies, and on the other hand, research can be conducted to design unique biomaterials to combine with the physiological process of apoptosis, and synergize with ABs to realize four‐dimensional therapies

These three engineering strategies correspond to the modifications made at different stages from AB production to application. The inherent heterogeneity of ABs makes it challenging to categorize them for studying. However, the presence of PS on their surface and “find‐eat” signals provide ABs with excellent targeting abilities toward phagocytic cells. Engineering aims to capitalize on strengths and address weaknesses by manipulating the membrane surface and contents of ABs and controlling their release to achieve high targeting, high loading rates, low biotoxicity, and sustainability, thus maximizing therapeutic effects. At present, few studies combine these three engineering methods, typically only focusing on one. This may be related to the insufficient comprehensive understanding of ABs. However, practical applications often require multi‐engineering to overcome the limitations of ABs from different angles.

For the future development of the engineering field of ABs, techniques that have been successful in exosomes, microvesicles, and liposomes can be introduced to ABs. Furthermore, innovative engineering strategies adapted to the unique properties of ABs can be explored beyond the successes of exosomes and microvesicles.

While ABs possess significant therapeutic potential, there remain challenges to be addressed:

1. The targeting ability of ABs depends mainly on its membrane proteins, but its membrane proteins have not yet been fully resolved, and a great deal of work is needed to refine the understanding of ABs' membranes to see whether there are other proteins besides PS that can be targeted to different cells, and whether these proteins are expressed on ABs of different cellular origins.
2. During apoptosis, the diameters of ABs tend to vary widely. Although the contents of ABs are currently thought to be randomly distributed during apoptosis, it is still unclear whether the composition of the contents of ABs would be related to the size of ABs, for example, whether larger ABs would have some organelles intact whereas smaller ABs would have only a small amount of cytoplasmic proteins and fragmented nuclear components. Figuring out these questions can help to categorize ABs in further detail and enable more precise medical treatment.
3. The separation of successfully modified ABs from those unmodified to obtain pure‐modified ABs is not yet achievable.
4. Many genetic materials from the parent cell present in ABs remain uncharacterized, and when utilized for disease treatment, these “impurities” may lead to unforeseen side effects. The problem of distinguishing which ABs are therapeutic and which are ineffective needs to be addressed. ABs should be reclassified and then applied rather than applying all ABs as a whole directly.

These challenges have undoubtedly hindered the research and application of ABs, and future research is urgently needed to address these issues and completely unleash the therapeutic potential of ABs.

## AUTHOR CONTRIBUTIONS

Zheyuan Hu and Shutong Qian conceived the idea; Zheyuan Hu wrote the manuscript; Qiuyu Zhao, Bolun Lu, Qian Lu, Yuhuan Wang, Liucheng Zhang and Xiyuan Mao participated in review and editing; Danru Wang and Wenguo Cui revised the manuscript; Xiaoming Sun participated in supervision.

## CONFLICT OF INTEREST STATEMENT

The authors declare no conflict of interest.
